# Dealing with Pheochromocytoma during the First Trimester of Pregnancy

**DOI:** 10.1155/2015/439127

**Published:** 2015-03-08

**Authors:** Konstantinos Kiroplastis, Apostolos Kambaroudis, Apostolos Andronikou, Andromachi Reklou, Dimitris Kokkonis, Panagiotis Petras, Apostolos Mamopoulos, Eudokia Anagnostara, Charalampos Spyridis

**Affiliations:** ^1^Fifth Surgical Department, Aristotle University of Thessaloniki, Hippokratio General Hospital of Thessaloniki, Konstantinoupoleos 49 Street, 54642 Thessaloniki, Greece; ^2^Second Propedeutic Department of Internal Medicine, Aristotle University of Thessaloniki, Hippokratio General Hospital of Thessaloniki, Konstantinoupoleos 49 Street, 54642 Thessaloniki, Greece; ^3^Third Obstetric Department, Hippokratio General Hospital of Thessaloniki, Konstantinoupoleos 49 Street, 54642 Thessaloniki, Greece; ^4^Anesthesiology Department, Hippokratio General Hospital of Thessaloniki, Konstantinoupoleos 49 Street, 54642 Thessaloniki, Greece

## Abstract

*Purpose*. Pheochromocytoma in association with pregnancy is a very rare, without specific symptoms, life-threatening condition, increasing both maternal and fetal mortality up to 50%. The present paper illustrates the case of a pregnant woman, diagnosed with pheochromocytoma, aiming to demonstrate and discuss the difficulties that arouse during the diagnosis and the problems concerning the treatment. *Patient*. A 34-year-old woman, in the 9th week of pregnancy, complained for headache, sweating, and a feeling of heavy weight on the right renal area. A tumor of 10 cm diameter at the site of the right adrenal was found. Twenty-four-hour urine catecholamine and VMA excretion levels were well raised. *Results*. Multidisciplinary approach treated the patient conservatively. Surgical resection of the tumor was performed after the 14th week of pregnancy at the completion of organogenesis. Neither postoperative complications occurred nor hypertension relapse was recorded. The fetus was delivered without complications at the 36th week. *Conclusions*. There are no consensus and guidelines for treating pheochromocytoma during pregnancy, especially when it is diagnosed in the first trimester. The week of pregnancy and a multidisciplinary approach will determine whether the pregnancy should be continued or not, as well as the time and the approach of surgical treatment.

## 1. Introduction

Definite treatment of pheochromocytoma is the surgical resection of the adrenal. Nowadays, in general population the laparoscopic adrenalectomy is the treatment of choice and is carried out as soon as diagnosis is made. However, when there is a concurrent pregnancy, the best treatment method has to take into consideration both mother and fetus, depending at a high extent on the severity of the symptoms, the gestational age, and patient's decision to continue the pregnancy or not, after being fully informed for potential risks [1–3].

A case of a pregnant patient we treated will be presented to discuss the difficulties of diagnosis and treatment that arise, needing individual solutions.

## 2. Case Report

A 34-year-old woman at the 9th week of her first pregnancy was admitted in the hospital, complaining of paroxysmal hypertension episodes for the last 8 months (2-3 monthly), lasting 1-2 minutes with maximum values of systolic blood pressure at 220–240 mmHg and diastolic blood pressure at 140 mmHg. The symptoms were followed by palpitation, headache, sweating, nonspecific gastrointestinal disorders, and a constant heavy weight on the right renal area.

Clinical examination of the abdomen was performed without any pathological findings.

**Figure 1:**
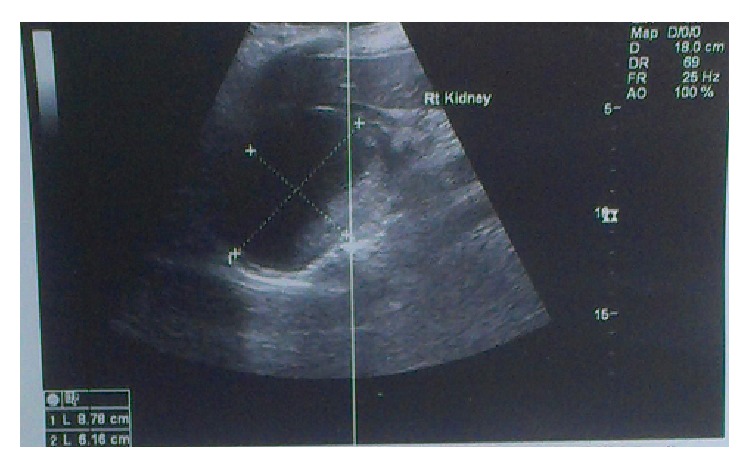
Ultrasound of the abdomen. Tumor between right kidney and liver, with mixed echogenicity, low vascularity, and dimensions 9 × 7.5 cm.

Abdominal ultrasound though showed a mass between the right kidney and the liver, with dimensions of 9 × 7.5 cm with mixed echogenicity and low vascularity (Figure 1).

**Figure 2:**
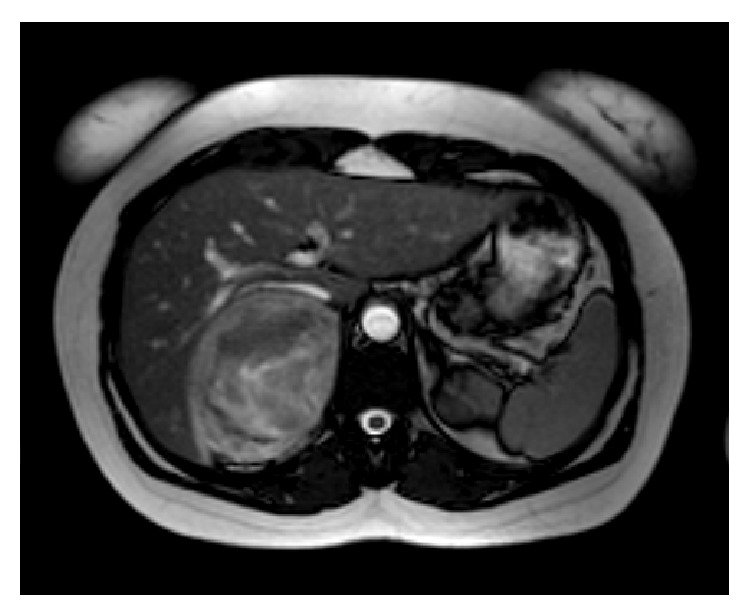
MRI describing mass of Dmax = 9 cm between right kidney and liver, with hemorrhagic elements.

Magnetic resonance imaging (MRI) confirmed previous findings. A well-defined, round mass of 9 cm maximum diameter, lateral to the aorta and between the right kidney and the liver, was described, displacing the right kidney. In T1-weighted images it had heterogeneous hypointensive signal relative to skeletal muscle with areas of isointensive signal, implying hemorrhagic areas (Figure 2). In T2-weighted images the mass was in some areas highly intense relatively to the liver (described as light-bulb bright), especially in the periphery, something typical of pheochromocytomas. Enhancement was heterogeneous, quick, and intense after contrast administrations. Neither extra-adrenal location was spotted nor lymph node metastasis.

24-hour urinary catecholamine excretion levels exceeded normal levels (889 mg/24 h) and so were vanillylmandelic acid (VMA) levels (28 mg/24 h) (Table 1). Increased aldosterone levels can be attributed to a secondary response to renin levels.

All the above led to the diagnosis of pheochromocytoma. Heart ultrasound was free of pathological findings and blood pressure Holter monitoring identified high values of both systolic (max 167 mmHg) and diastolic (max 109 mmHg) blood pressure. The workup included ultrasound of the fetus, which was normal.

A multidisciplinary meeting was held, in the presence of anesthesiologist, gynecologist, internal medicine specialist, and general surgeon, in order to determine the best treatment plan. Until the end of the first trimester of pregnancy when fetal organogenesis would be completed, alpha- and beta-blockers (terazosin and atenolol) were used to treat hypertension. Terazosin was administered in a gradually increased final dose of 5 mg twice a day and 25 mg once per day of atenolol was prescribed a couple of days later. 24-hours of blood pressure monitoring followed, recording a maximum measurement of 179/105 mmHg and a minimum of 117/76 mmHg (mean blood pressure 122/83 mmHg). Heart rate ranged between 70 and 100 heart beats per second with a mean of 82. As a result, blood pressure was considered well controlled with medication and at the 14th week of pregnancy the patient was admitted to the surgical department. Preoperative administration of crystalloids and fresh frozen plasma was done, to increase endovascular volume. An epidural catheter was placed for postoperative analgesia, a central intravenous line for the measurement of the central venous pressure, two peripheral vein catheters for the perioperative administration of fluids and drugs, arterial line for determination of air blood gases, a urine catheter to monitor urination, and a warming blanket to prevent hypothermia. Intravenous administration of 1.5 gr of cefuroxime followed and the patient was intubated with general anesthesia with cisatracurium besylate, etomidate, and fentanyl. Before intubation, esmolol was administrated to prevent tachycardia. To retain general anesthesia during the surgery sevoflurane was administrated. The anesthetic drugs used were the ones with the less adverse effects in pregnancy, while providing an absolutely sufficient (deep) level of anesthesia and muscle relaxation, in order to avoid uncontrolled excretion of catecholamines.

**Figure 3:**
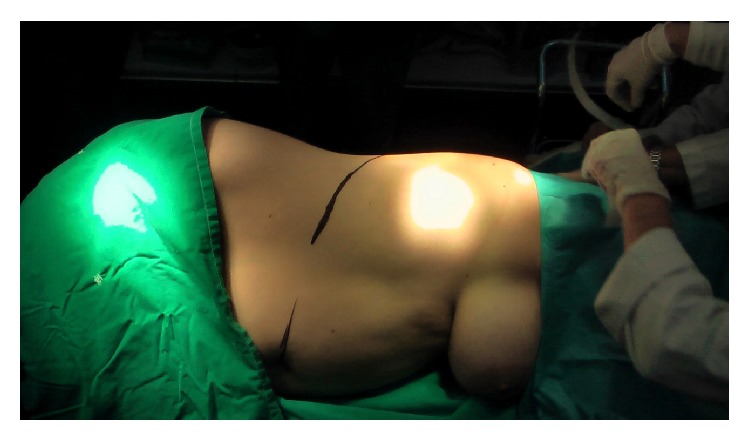
Patient at left side, posterolateral extraperitoneal incision was made to approximate the tumor.

The patient lied at the left side and a posterolateral extraperitoneal incision was made to approximate the tumor (Figure 3).

**Figure 4:**
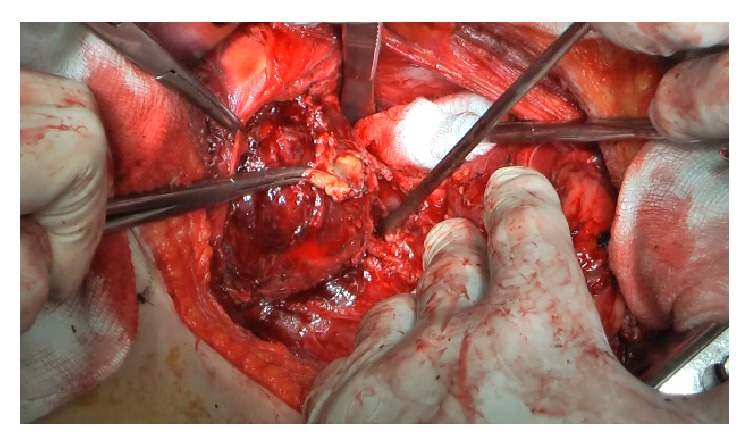
Quick dissection and ligation of right adrenal vein.

**Figure 5:**
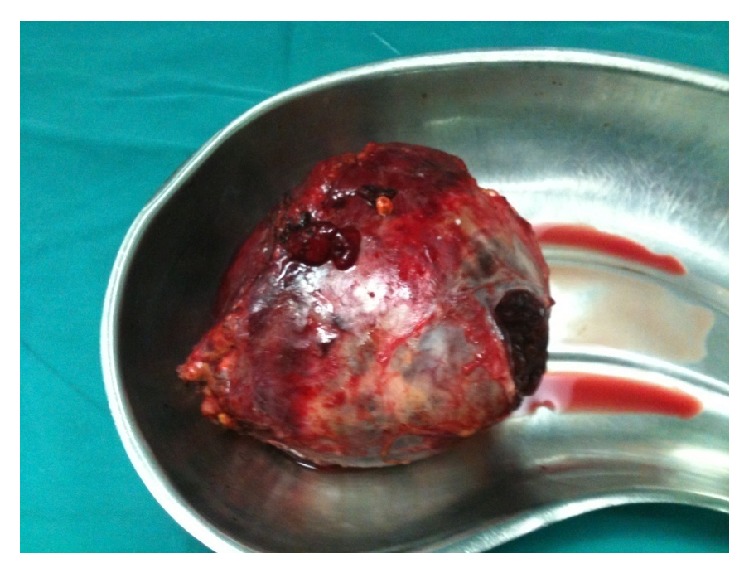
Right adrenalectomy.

The right adrenal vein was identified and immediately ligated (Figure 4), with minimal tumor manipulation. Afterwards, right adrenalectomy was carried out, without remarkable blood pressure fluctuations (maximum systolic blood pressure: 158 mmHg) (Figure 5). The use of sodium nitroprusside was not needed.

**Figure 6:**
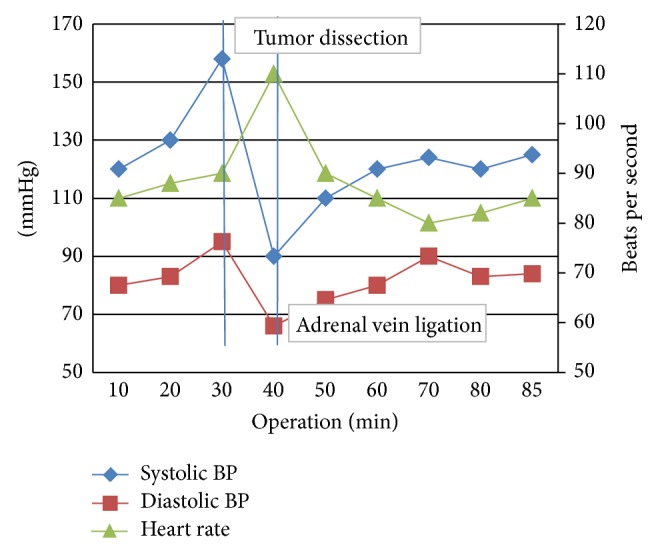
Cardiovascular responses during surgery.

After tumor resection, there was a drop of blood pressure, with a minimum value of 100 mmHg that was treated with intravenous administration of fluids. Blood pressure fluctuation during the surgery is presented (Figure 6). The duration of the surgery was 85 minutes in which 450 mL of urines was produced and the estimated blood loss was no more than 150 mL. Hence, there was no need for blood transfusion.

Anesthesia's awakening was without complications and the postoperative analgesia was based on the administration of ropivacaine via the epidural catheter. Additionally, no need for vasopressors was recorded.

At the end of the procedure, fetal ultrasound was performed by the gynecologist, without abnormal findings. Medication for hypertension was ceased, without hypertension relapse (maximum systolic blood pressure measured at 120 mmHg), and urine catecholamine excretion levels were at normal values at postoperative followup. Postoperative course was smooth and uncomplicated, with regular ultrasound examinations of the fetus. The patient was discharged at the 5th postsurgical day. The rest of the pregnancy was normal, without fetal developmental problems and without hypertension relapse. At the 36th week of pregnancy, the patient gave birth to a healthy neonate without any occurring difficulties or hypertension episodes.

## 3. Discussion

Hypertension in pregnancy is defined as a raised blood pressure over 140/90 mmHg in a woman with lower measured values 20 weeks before. It includes the following conditions: (a) chronic hypertension that exists before pregnancy or develops before the 20th week of gestation; (b) preeclampsia and eclampsia; (c) chronic hypertension with development of preeclampsia; (d) and gestational hypertension that develops after the 20th week of gestation and abates after labor.

Hypertension can be accompanied by proteinuria, low number of platelets, severe headache that is frontal and throbbing, sudden weight gain, nausea, right upper quadrant abdominal tenderness, rapidly increasing extremities edema and weight gain, visual disorders, and lung or kidney dysfunction. In all these cases, preeclampsia is diagnosed. When untreated, eclampsia is established and the patient may present seizures with high mortality rate. Diabetes, obesity, African origin, family or personal previous history, preexisting kidney disease and hypertension, multiple fetuses, and maternal age below 20 and over 40 years old are conditions of increased risk for developing preeclampsia [4]. Nowadays there is a screening algorithm developed predicting early preeclampsia [5]. Preeclampsia is rare before the 3rd trimester. In cases developed earlier, exclusion of gestational trophoblastic disease and molar pregnancy is necessary. Pheochromocytoma is a rare tumor of the chromaffin cells. It is diagnosed at the 0.1–0.6% of the hypertensive population [6]. It is even more uncommon when associated with pregnancy (0.002–0.007%). When diagnosis is made before labor, mortality is at 0–2% and 11–15% for mother and fetus, respectively [2]. Despite the increased knowledge and diagnostic tools, almost 20% of the cases remain undiagnosed until labor, leading to a remarkable increase of maternal and fetal mortality (58% and 56%, resp.). The high levels of mortality are related to complications of a hypertensive crisis like stroke and cardiac arrest, pulmonary edema, placenta abruption, fetal hypoxia, and intrauterine growth retardation [3, 6, 7]. Causes for delayed diagnosis are the rarity of the disease, nonspecific symptoms appearing to numerous other conditions, and the absence of symptoms, even in advanced pregnancy [3].

The classic triad of symptoms for pheochromocytoma consisting of headache, palpitation, and sweating, coming along with a hypertensive crisis, is not always present. Other symptoms such as flushing and tremor may be referred. It is of significant importance to clarify that hypertension due to pheochromocytoma is paroxysmal, appearing before the 20th week of pregnancy and it may be followed by orthostatic hypotension, unlike gestational hypertension.

Catecholamine release can occur from pressure on the tumor from the expanding uterus, uterine contractions, and fetal movements or during labor causing hypertensive crises. When there are edema, proteinuria, and increased levels of uric acid, the presence of pheochromocytoma is unlikely [2, 6–8]. The presented patient has been having paroxysmal hypertensive episodes for the last 8 months, before pregnancy, followed by palpitation, headache, sweating, nonspecific gastrointestinal disorders, and constant sense of heavy weight at the right renal area. A hypertensive crisis led her to be admitted in a hospital with expertise in adrenal disorders.

Because pregnancy ionizing radiation cannot be applied, computed tomography (CT) and 123I-Meta-Ido-Benzo-Guanidine (MIBG) are contraindicated. Estimation of twenty-four-hour urine catecholamine and VMA excretion can be applied. Imaging with ultrasound followed by MRI contributes to definite diagnosis, as happened in the presenting case.

The only definite treatment is the surgical excision of pheochromocytoma. However, the ideal treatment is directly related with the gestational age. Elective alpha-blockers are at the first line for medical treatment. Even though they pass the placenta, they are used with safety in obstetrics. Most of the time, phenoxybenzamine (a1 and a2 antagonist), doxazosin (eclectic a1 inhibitor), and prazosin are used. In acute conditions phentolamine and sodium nitroprusside can be administrated. Reported cyanide toxicity may be low with low flow administration (<1 *μ*g/kg/min) best to be completely avoided. After a couple of days of alpha-blockers administration, beta-blockers are added to the regiment (propranolol and atenolol). Furthermore, it is of great importance to increase the endovascular volume in pregnant women preoperatively, administrating intravenous fluids and salt, in order to eliminate the possibility of serious postoperative hypotension [6–8]. Our patient's blood pressure was well-controlled by administration of alpha- and beta-blockers, until the first trimester of pregnancy was completed and adrenalectomy was carried out afterwards.

Time and type of surgical approach is controversial, as there are no guidelines and the number of cases is very small to have definitive experience. It depends on individual center's practice preferences and tumor and uterus size. Open adrenalectomy either through laparotomy or posterolateral extraperitoneal access was the method of choice. However, following the growing experience in minimal invasive surgery (laparoscopic or robotic assisted adrenalectomy), nowadays it appears to have better results compared to the open approach, especially regarding catecholamine level rise during the procedure. As a result, it is safer for both mother and fetus. Complication rate is lower than 8% and as a result laparoscopic approach tends to be the widely chosen surgical approach [2, 6, 9, 10]. Our center has experience in both laparoscopic and open approach. Due to the border line size of the pheochromocytoma (6 cm) open approach was chosen as the safest option for this patient.

The time of surgical treatment depends on a number of factors, such as gestational age, response to conservative treatment, tumor accessibility, and signs of fetal stress [2, 8, 10]. Some authors in international literature support the immediate termination of pregnancy and patient preparation for tumor excision later on. To defend their opinion, they pose the doubtful long-term response of the conservative treatment and the possibility of malignancy [2, 7]. There is a recent trend in the last years to excise the tumor without terminating the pregnancy, when diagnosis is set before the 24th week of pregnancy. In those cases that diagnosis is made after the 24th week of pregnancy, effort is made to conservatively control symptoms with medication, until fetus is viable and at that point both tumor excision and caesarian labor take place [2, 6, 7]. When diagnosis is set early in the first trimester of pregnancy, usually termination of gestation is suggested, because there is a very high risk of abortion. The lower rate of spontaneous abortion is at the end of the first trimester (15%) and it gets even lower, up to 0% at the second trimester of pregnancy, followed by a lower rate of premature birth of 5% [6, 7, 9, 11–13]. Regarding the presented case, the diagnosis was set at the end of the first trimester of pregnancy. At the multidisciplinary meeting board and after patient's consensus, surgical approach was decided to take place at the 14th week of pregnancy, in order for fetal organogenesis to be fully completed.

After tumor excision, blood pressure may remain high or even relapse at the 50% of the patients. This phenomenon is attributed to the extra-adrenal (30%) and adrenal (14%) sites [6]. For that reason, patients must be followed up after the operation in a yearly basis. In cases of genetic predisposition or heritability, level of suspicion must be particularly high [2, 6, 10, 14, 15]. Our patient one year after the operation is free of symptoms with blood pressure at normal values (135/85 mmHg).

The approach of fetal delivery is crucial. In case the tumor is not excited, a vaginal delivery is very likely to cause catecholamine excretion due to travails, extruding contractions, and further management. In these cases, fatal complications rate is up to 31%, in contrast to 19% of the cesarean labor [2, 6, 8, 14]. It is for that reason why caesarean delivery is preferred approach with lower risk for patients that tumor have not been excised yet. On the contrary, if the tumor has been excised earlier, vaginal labor is equally safe [2, 6]. As far as the aforementioned patient is concerned, a vaginal labor without any perinatal complications was carried out.

## 4. Conclusion

- The rarity of the condition and the limited number of recorded cases do not allow space for definitive conclusions and their generalization.
- The severity of the condition is obvious, by the high levels of fetal and maternal mortality, when early diagnosis is missed. The fact that there are no specific signs and symptoms, and patient's clinical condition is common to a number of other diseases, such as gestational hypertension, and makes differential diagnosis even harder. As a result, high level of suspicion is necessary.
- The restrictions on imaging procedures increases the risk of missed extra-adrenal sites, which is why genetic predisposition control is needed.
- Surgical excision of the tumor is the definite chosen treatment nowadays. The second trimester of pregnancy appears to be the safest time to operate, in cases diagnosis is set before the 24th week of pregnancy and after organogenesis completion.
- Minimal invasive surgery (laparoscopic or robotic assisted adrenalectomy) is considered safe when applicable.
- In cases pheochromocytoma is diagnosed beyond the 24th week of pregnancy, conservative treatment with medication is applied, in order to control the symptoms, until fetus is considered viable. Caesarian labor follows and synchronous or metachronous tumor excision is carried out. It is of great importance and necessity that treatment plan is decided through a multidisciplinary board meeting, taking into consideration each case individually, for benefit of both mother and fetus.

## Figures and Tables

**Table 1:** Patient's adrenal hormone levels.

	Value	Normal values
24 h-urine VMA	28.0	1.8–6.7 mg/24 h
24 h-catecholamines	889	14–108 *μ*g/24 h
Serum aldosterone	59.0	4.0–31 ng/dL (standing)
Serum cortisol	19.1	6.2–19.4 *μ*g/dL (morning)
Serum dehydroepiandrosterone DHEA	2.8	1.0–10.5 ng/mL

## Abbreviations

MRI: Magnetic resonance imaging
VMA: Vanillylmandelic acid
CT: Computed tomography
MIBG: 123I-Meta-Ido-Benzo-Guanidine.
